# Liposome Encapsulation Enhances the Antidiabetic Efficacy of Silibinin

**DOI:** 10.3390/pharmaceutics16060801

**Published:** 2024-06-13

**Authors:** Svetlana Dinić, Jelena Arambašić Jovanović, Aleksandra Uskoković, Aleksandra Jovanović, Nevena Grdović, Jovana Rajić, Marija Đorđević, Ana Sarić, Branko Bugarski, Melita Vidaković, Mirjana Mihailović

**Affiliations:** 1 Department of Molecular Biology, Institute for Biological Research “Siniša Stanković”—National Institute of the Republic of Serbia, University of Belgrade, 11108 Belgrade, Serbia; jelena.arambasic@ibiss.bg.ac.rs (J.A.J.); auskokovic@ibiss.bg.ac.rs (A.U.); nevenag@ibiss.bg.ac.rs (N.G.); jovana.rajic@ibiss.bg.ac.rs (J.R.); marija.sinadinovic@ibiss.bg.ac.rs (M.Đ.); ana.saric@ibiss.bg.ac.rs (A.S.); melita@ibiss.bg.ac.rs (M.V.); mista@ibiss.bg.ac.rs (M.M.); 2 Institute for the Application of Nuclear Energy INEP, University of Belgrade, 11080 Belgrade, Serbia; ajovanovic@inep.co.rs; 3 Faculty of Technology and Metallurgy, University of Belgrade, 11000 Belgrade, Serbia; branko@tmf.bg.ac.rs

**Keywords:** diabetes, insulin, silibinin, liposomes, antidiabetic, anti-inflammatory, C-reactive protein, collagen deposition

## Abstract

Silibinin has considerable therapeutic potential for the treatment of diabetes through anti-inflammatory, antioxidant, and immunomodulatory properties. However, the therapeutic application of silibinin is quite limited due to its poor bioavailability. In the present study, an attempt was made to improve the antidiabetic efficacy of silibinin by its encapsulation in liposomal vesicles. The liposomes with a high encapsulation efficiency of silibinin (96%) and a zeta potential of −26.2 ± 0.6 mV were developed and studied using nicotinamide/streptozotocin-induced diabetic rats. Administration of silibinin-loaded liposomes to diabetic rats lowered glucose levels, increased insulin levels, and improved pancreatic islet architecture. The anti-inflammatory effect of silibinin-loaded liposomes was demonstrated by a decrease in serum C-reactive protein (CRP) levels and a reduced deposition of collagen fibers in the islets of diabetic rats. Furthermore, silibinin-loaded liposomes were more efficient in lowering glucose, alanine transaminase, triglyceride, and creatinine levels in diabetic rats than pure silibinin. In addition, silibinin-loaded liposomes had a significantly better effect on beta-cell mass and Glut2 glucose receptor distribution in diabetic islets than pure silibinin. The present results clearly show that liposome encapsulation of silibinin enhances its antidiabetic efficacy, which may contribute to the therapeutic benefit of silibinin in the treatment of diabetes and its complications.

## 1. Introduction

Diabetes mellitus is one of the four most common non-communicable diseases. There are currently 540 million people living with diabetes worldwide, and it is estimated that the number of diabetes patients will increase by 2045 to around 783 million patients [1]. Type 1 diabetes (T1D) affects about 10% of diabetes patients, mostly children and adolescents, while type 2 diabetes (T2D) occurs in 85–90% of diabetes patients, usually older than forty years, but is increasingly found in younger people [2]. T2D is usually a combination of pancreatic beta-cell dysfunction and insulin resistance (IR) [3]. In response to IR, which is generally associated with obesity, metabolic disorders, or aging, pancreatic islet cells increase their cell mass and insulin secretion. If the functional increase in beta-cell mass does not compensate for the extent of IR and insulin deficiency, glucose homeostasis is disrupted, and T2D develops. The main mechanisms thought to lead to IR and beta-cell dysfunction in T2D are oxidative stress, lipotoxicity, glucotoxicity, endoplasmic reticulum stress, and autoimmune responses [2,4]. All of these factors are considered to either induce an inflammatory response or to be associated with or intensified by inflammation [3]. Fibrosis, which is known to be a hallmark of chronic inflammation, has also been found in islet sections from T2D patients [5]. In addition, prospective and cross-sectional studies have reported elevated levels of cytokines, chemokines, acute-phase proteins such as C-reactive protein (CRP), haptoglobin, and fibrinogen in the circulation of T2D patients [6,7]. In view of the above, chronic, low-grade inflammation in islet cells is considered a hallmark of T2D [3].

Despite a wide range of available antidiabetic drugs, less than half of diabetic patients achieve optimal blood glucose control [8]. On the other hand, good glycemic control as such is no guarantee for the prevention of long-term microvascular (nephropathy, retinopathy, and neuropathy) or macrovascular (myocardial infarction, heart failure, stroke, and peripheral arterial disease) complications or liver diseases [3,9]. This underlines the importance of developing new or improving existing therapeutic strategies for diabetes management. Flavonolignan silibinin is the main component of the standardized silymarin extract derived from the seeds of milk thistle *Silybum marianum* [10]. Silymarin and silibinin have shown considerable therapeutic potential through anti-inflammatory [10], antioxidant [11], antiviral [12], immunomodulatory [13], and anticancer properties [14]. Silymarin and silibinin are traditionally used for the treatment of gallbladder and liver diseases due to hepatoprotective properties [15]. Additionally, numerous experimental studies showed antidiabetic effects of silibinin, such as the normalization of glucose levels in diabetic animals, the increase of insulin levels via increased insulin sensitivity in liver and muscle cells, and the improvement of pancreatic beta-cell function [16,17]. Clinical studies investigating the efficacy of silymarin in diabetic patients showed improved glucose homeostasis and a reduction in inflammation and oxidative stress [18]. Although silibinin is considered a safe compound for therapeutic applications with minimal side effects, its bioavailability is limited due to its poor water solubility, instability, and low intestinal absorption [19].

The functionality, stability, and bioavailability of bioactive plant compounds, including silibinin, depend on various factors such as temperature, irradiation, light, enzymes, pH, free radicals, and storage conditions. Therefore, the main advantages of encapsulating these sensitive bioactives in different carriers are improved stability during storage, protection from environmental and/or gastrointestinal conditions, better bioavailability, and controlled and/or prolonged release [20]. To date, various delivery systems have been explored to improve the water solubility and efficacy of orally administered phytoconstituents [19]. Liposomal vesicles represent a revolutionary class of drug delivery systems that can transport therapeutic payloads with precision and efficiency. The unique composition of these particles not only enables controlled release and targeted delivery but also increases bioavailability and biocompatibility, making liposomes a disruptive force in modern medicine [20,21,22]. Due to their amphiphilic nature, liposomal vesicles are able to encapsulate both lipophilic and hydrophilic components [23,24]. Furthermore, the use of naturally readily abundant phospholipids in the preparation of liposomal carriers makes them biocompatible and cost-effective delivery systems. Phospholipids in liposomal particles do not trigger a reaction with taste receptors, making liposomes a suitable carrier to mask even the unpleasant taste of polyphenols [25].

Improving the intelligent dissolution and consistent delivery of phenolic compounds is a current challenge that is receiving considerable research attention in order to align it with desired therapeutic goals. Utilizing existing knowledge on liposomal vesicle design appears to be a promising way to address this challenge [23,26]. This study aimed to investigate whether the antidiabetic efficacy of silibinin can be improved by its formulation in liposomal vesicles. To that end, silibinin-loaded liposomes were prepared and characterized and then tested for antidiabetic efficacy in the T2D animal model. The study examined biochemical parameters associated with diabetes, factors reflecting liver and kidney function, as well as pancreatic tissue morphology and functionality. Additionally, indicators of inflammation were analyzed as part of the study.

## 2. Materials and Methods

### 2.1. Chemicals

Silibinin (≥98% HPLC grade), nicotinamide (NA, ≥98% HPLC grade), streptozotocin (STZ), Tween 20, and 3,3′-diaminobenzidine (DAB) were purchased from Sigma-Aldrich (St. Louis, MO, USA). Phospholipon 90G (phosphatidylcholine from soybean) was purchased from Natterman Phospholipids (Köln, Germany) and ethanol (Absolute, Extra Pure) from Fisher Scientific (Loughborough, UK). Distilled water was purified through a Simplicity UV^®^ water purification system (Merck Millipore, Merck KGaA, Darmstadt, Germany).

### 2.2. Liposomal Preparation

Silibinin-loaded liposomes were prepared using the proliposome procedure previously described [27]. Namely, a mentioned technique for liposomal preparation is described with the aim of avoiding the use of pharmaceutically unacceptable solvents and reagents (such as chloroform used for obtaining the thin film of lipids and active compounds in the thin-film method), as well as energy-expensive procedures, e.g., sonication. The procedure is based on the initial preparation of a proliposome mixture containing lipids or phospholipids, ethyl alcohol, and water, which is converted to liposomes by a simple dilution step. Hence, the proliposome method represents a simple and one-step procedure that does not require complex or expensive devices and complicated/multistage processes. Therefore, the procedure was performed in the following way: (1) 43.75 g of phospholipids (fatty flakes of phosphatidylcholine from soybean, commercially available mixture of phospholipids named Phospholipon 90G) were measured in a beaker; (2) 4.375 g of silibinin (off white to light yellow powder, previously stored at 4 °C) was measured in a beaker and dissolved using 50 mL of ethanol (measured in a laboratory graduated cylinder); (3) dissolved silibinin in ethanol was transferred in the mixture of phospholipids previously measured in a beaker and mixed; (4) all mixture was heated to 60 °C and stirred for 30 min on the laboratory magnetic stirrer with PT 1000 temperature sensor (IKA RCT basic, IKA, Staufen, Germany) using a stir bar (small white oblong Teflon-covered magnet) at a speed of 500 rpm; due to the presence of ethanol and high temperature, phospholipids were dissolved, and a molten mixture was obtained; (5) the heated beaker with the mixture was uncovered to allow the evaporation of ethanol; (6) after the evaporation of ethanol, the sample was cooled to 25 °C yielding a proliposome mixture; and (7) 350 mL of ultrapure water (measured in a laboratory graduated cylinder) was added in small portions in order to produce liposome particles, i.e., the proliposome mixture was converted to a liposomal dispersion due to the slow addition of water. Subsequently, the mixture was stirred in a covered beaker for 2 h at 800 rpm and at room temperature on the laboratory magnetic stirrer with a temperature sensor using a stir bar. The temperature was controlled because of the potential heating of the magnetic stirrer and, consequently, the sample because of the long stirring time. In the previous work [26], different ratios of silibinin, phospholipids, and water were used but, in that case, significantly lower encapsulation efficiency was determined. Plain liposomes (without silibinin) were prepared as a control. Namely, phospholipids and ethanol were mixed and heated to 60 °C for 30 min to obtain a molten mixture and, after that, to evaporate ethanol. After cooling down to 25 °C, ultrapure water was added in small portions with the aim of hydrating the phospholipid mixture and forming liposomal vesicles. The liposomes were stored at 4 °C until further use.

### 2.3. Determination of the Encapsulation Efficiency

Free silibinin was removed from liposomal suspension by centrifugation at 17,500 rpm for 45 min at 4 °C in a Thermo Scientific Sorval WX Ultra series ultracentrifuge (Thermo Scientific, Waltham, MA, USA). The quantity of silibinin in the supernatant was determined spectrophotometrically at 280 nm using a UV Spectrophotometer UV-1800 (Shimadzu, Kyoto, Japan). Encapsulation efficiency (EE%), i.e., the amount of silibinin encapsulated in liposomes, was calculated as the amount of silibinin quantified in the supernatant divided by the amount of silibinin used for the liposomal formulation:

EE (%) = (m_i_ − m_s_)/m_i_ × 100,

where m_i_ is the initial content of silibinin used for obtaining silibinin-loaded liposomes, and m_s_ is the content of silibinin quantified in the supernatant.

### 2.4. Liposomal Characterization

Liposomal characterization was also performed via determination of the vesicle size, polydispersity index (PDI), zeta potential, and mobility of silibinin-loaded liposomes using photon correlation spectroscopy in Zetasizer Nano Series, Nano ZS with the measurement range of 0.6 nm to 6 mm (Malvern Instruments Ltd., Malvern, UK). Photon correlation spectroscopy, also known as dynamic light scattering, is extensively employed in liposomal vesicle size distribution analysis by measuring the time-dependent fluctuation of light scattered from vesicles experiencing Brownian motion. The method is frequently performed to assess zeta potential as well. Namely, the sample was diluted 500 times using ultrapure water. One milliliter of diluted liposome dispersions was placed in 3 mL disposable 12 mm square polystyrene cuvettes (DTS0012), and the particle size and PDI were measured three times at 25 °C. Disposable capillary cells constructed from polycarbonate with gold-plated electrodes (DTS1060) and 850 µL of the sample were used for zeta potential and mobility measurements. Malvern Dispersion Technology Software (DTS), version 7.03 (Malvern Instruments Ltd., Malvern, UK) was also employed with multiple narrow mode (high resolution) data processing, and mean values for size (nm), PDI, zeta potential (mV), and mobility (µmcm/Vs) and standard deviation values are presented.

### 2.5. Animals

Male Wistar albino rats, aged 2.5 months and weighing 220–250 g, were kept under standard laboratory conditions of relative humidity, temperature, and light/dark cycle and were allowed free access to a standard chow diet. The study was conducted according to the guidelines of Directive 2010/63/EU on the protection of animals used for experimental and other scientific purposes and approved by the Ethical Committee for the Use of Laboratory Animals of the Institute for Biological Research “Siniša Stanković”—National Institute of the Republic of Serbia, University of Belgrade based on the approval of the Ministry of Agriculture, Forestry and Water Management—Veterinary Administration (protocol code 323-07-06404/202205; date of approval 1 June 2022).

### 2.6. Experimental Design

T2D was induced in the overnight fasting male Wistar albino rats by intraperitoneal (i.p.) injections of NA (150 mg/kg b.w.) dissolved in 0.9% saline and followed (after 15 min) by i.p. injection of STZ (65 mg/kg b.w.) dissolved just before use in 0.05 M sodium citrate (pH 4.5). This step was repeated 1 day later [28,29]. In this well-established experimental animal model of T2D, NA protects pancreatic beta-cells from STZ toxicity, resulting in relative insulin deficiency [30]. The described treatment did not cause animal death. The blood glucose level was measured 24 h after the last STZ injection (blood samples were collected from the tail vein). Rats with fasting blood glucose levels exceeding 10 mmol/L were considered diabetic. Glucose was measured with a blood glucometer (Accu-Chek Active, India). The rats were randomly divided into seven groups as follows: (1) non-diabetic group (C, n = 7); (2) non-diabetic group receiving an equivalent volume of plain liposomes (without silibinin) suspended in water daily by oral gavage for 4 weeks (C/L, n = 7); (3) non-diabetic group receiving silibinin (50 mg/kg b.w.) suspended in 1% carboxymethyl-cellulose (CMC), daily by oral gavage for 4 weeks (C/SB, n = 7); (4) non-diabetic group receiving silibinin-loaded liposomes equivalent to 50 mg/kg of silibinin, daily by oral gavage for 4 weeks (C/LSB, n = 7); (5) diabetic group (D, n = 8); (6) diabetic group receiving silibinin suspended in 1% CMC (50 mg/kg b.w.), daily by oral gavage for 4 weeks (D/SB, n = 8); (7) diabetic group receiving silibinin-loaded liposomes equivalent to 50 mg/kg of silibinin, daily by oral gavage for 4 weeks (D/LSB, n = 8). After 4 weeks, the animals were fasted overnight and sacrificed by euthanasia using a guillotine, according to all recommendations of the guidelines for euthanasia of animals. Death comes quickly without any suffering of animals. Euthanasia was performed on animals one by one (in the block separated from the experimental room where the animals were held in order to avoid the stress of the animals) by well-trained staff. Immediately before sacrifice, the blood was collected from the tail vein of the animal for glucose measurement and for preparation of serum, which was stored at −20 °C until use. After blood collection and sacrifice, an abdominal incision was made, and the tail part of the pancreas was removed and fixed in 4% buffered formalin for histological examinations.

### 2.7. Determination of Biochemical Parameters of Diabetes

Serum, used for the determination of biochemical parameters, was collected after blood clotting and centrifugation at 2000× *g* for 10 min. Blood glucose levels in all experimental groups were verified at the beginning of the experiments (24 h after administration of the last dose of STZ) and the end of treatment (24 h after the last dose of plain liposomes, silibinin, or liposomal encapsulated silibinin) using a commercial kit (Gluco-quant Glucose/HK; Boehringer Mannheim, Mannheim, Germany). Hemoglobin (Hb) and glycosylated hemoglobin (GlyHb) were determined as described previously [31,32]. Serum triacylglycerol was measured with an enzymatic kit (Randox Laboratories, Crumlin, UK). Serum creatinine concentrations were determined using Cayman’s Creatinine Assay according to the manufacturer’s instructions. Activities of ALT (alanine transaminase) and AST (aspartate transaminase) were estimated by measuring the produced oxaloacetate and pyruvate, respectively, using an optimized standard UV kinetic method kit (GPT (ALAT) IFCC mod; GOT (ASAT) IFCC mod). Blood urea nitrogen (BUN) level was estimated according to the GLDH method (Human, Wiesbaden, Germany). CRP levels were determined using an immunoturbidimetric method [33].

### 2.8. Histological and Immunohistochemical Examination of the Pancreas

The tail part of the pancreas (in close proximity to the spleen) excised from Wistar rats in all experimental groups was fixed in 4% formalin. After 24 h fixation, all tissues were washed in tap water overnight and then dehydrated in ascending alcohol series up to 100%, cleared in xylene, and embedded in paraffin wax. Paraffin blocks were then sectioned at 5 μm thickness using a microtome. Formalin-fixed, paraffin-embedded tissue sections placed on superfrost microscope slides were treated with xylene to remove the paraffin and rehydrated through graded concentrations of ethanol in water, with the final rinse in pure water. Pancreatic tissue sections, prepared in that way, were used for histological and immunohistochemical examination. For histological analysis, tissue sections were stained with hematoxylin and eosin, and for collagen fiber detection, tissue sections were stained with Masson trichrome stain. After staining, sections were mounted and observed with a bright field microscope (Leica DMLB; objective magnification 20×). For immunohistochemical analysis, deparaffinized and rehydrated pancreas sections were incubated in 0.3% hydrogen peroxide/methanol for 20 min to block endogenous peroxidase. After washing in PBS (phosphate-buffered saline), heat-induced antigen retrieval was performed in 0.01 M sodium citrate. After blocking in 5% bovine serum albumin, the primary antibody was applied overnight at +4 °C. Monoclonal antibody raised against insulin and polyclonal antibodies raised against glucagon and GLUT2 (Santa Cruz Biotechnology, Santa Cruz, CA, USA) were diluted 1:300, 1:200, and 1:75, respectively. After washing, sections were incubated one hour at room temperature with appropriate horseradish peroxidase (HRP)-conjugated secondary antibody (anti-mouse-1:200; anti-rabbit-1:200; Santa Cruz Biotechnology, Santa Cruz, CA, USA) and afterward stained with 3,3′-diaminobenzidine (DAB), counterstained with hematoxylin, mounted and observed under a light microscope (Leica DMLB; objective magnification 20× for insulin and glucagon and 40× for GLUT2).

### 2.9. Statistical Analysis

The data were expressed as the mean ± SD (standard deviation). For intergroup comparison between two means, first, a one-way Analysis of Variance (ANOVA) was used, and in case of significance, a Tukey’s multiple comparison test was applied. The difference was considered statistically significant at *p* < 0.05.

## 3. Results

### 3.1. Encapsulation Efficiency, Vesicle Size, PDI, Zeta Potential, and Mobility of Silibinin-Loaded Liposomes

To characterize the silibinin-loaded liposomes, the encapsulation efficiency, particle size, PDI, zeta potential, and mobility were determined immediately after the preparation of the liposomes (Table S1, Supplementary Material). The encapsulation efficiency of silibinin in phospholipid liposomes was >96% (Table S1, Supplementary Material). As shown in Table S1, the liposome size was 2024.7 ± 22.1 nm, while the PDI as a measure of vesicle size distribution was 0.323 ± 0.025. The zeta potential, a measure of system stability, showed a negative value and amounted to −26.2 ± 0.6 mV, whereas the mobility, as a function of size, zeta potential, and lipid composition, was −2.06 ± 0.05 µmcm/Vs (Table S1, Supplementary Material).

### 3.2. Effects of Silibinin and Silibinin-Loaded Liposomes on Glucose, Glycated Hemoglobin, and Insulin Levels in Non-Diabetic and Diabetic Rats

**Figure 1:**
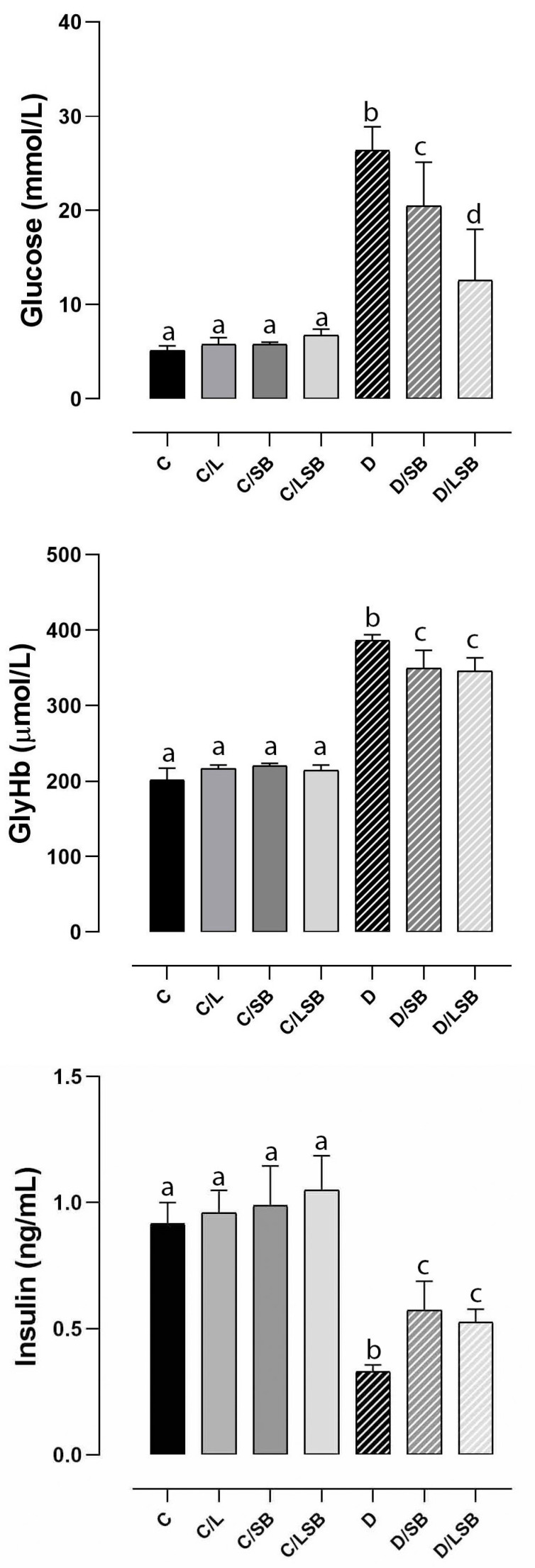
Effects of silibinin and silibinin-loaded liposomes on serum levels of glucose, glycated hemoglobin (GlyHb), and insulin in non-diabetic and diabetic rats. C—non-diabetic group; C/L—non-diabetic group treated with liposomes; C/SB—non-diabetic group treated with silibinin; C/LSB—non-diabetic group treated with silibinin-loaded liposomes; D—diabetic group; D/SB—diabetic group treated with silibinin; D/LSB—diabetic group treated with silibinin-loaded liposomes. Results are expressed as means ± SD (N = 7 for non-diabetic groups; N = 8 for diabetic groups). Means followed by different letters (a, b, c, d) indicate significant differences at *p* < 0.05 (one-way ANOVA followed by Tukey’s multiple comparison test).

As depicted in Figure 1, the blood glucose concentration in the diabetic rats (D) increased fivefold compared to the control rats (C). Treatment of the diabetic rats with silibinin (D/SB) or with silibinin-loaded liposomes (D/LSB) significantly lowered glucose levels compared to the D group (by 1.3-fold and 2-fold, respectively) (Figure 1). However, treatment with silibinin-loaded liposomes (D/LSB group) exhibited a more significant downregulation of blood glucose concentration compared to effects obtained in rats treated with pure silibinin (D/SB group). In the non-diabetic groups treated with liposomes (C/L), pure silibinin (C/SB), or silibinin-loaded liposomes (C/LSB), there was no difference in glucose levels compared to the control group (C) (Figure 1). Non-enzymatic protein glycation, influenced by the increase in blood glucose levels, was assessed by GlyHb level, which was 1.9 times higher in diabetic condition (D) than in the C group (Figure 1). The GlyHb levels in both D/SB and D/LSB were 1.1 times lower than in the D group. The GlyHb values in C/L, C/SB, and C/LSB did not differ from those in the C group. The insulin level decreased by almost 2.8-fold in the D group compared to the C group (Figure 1). Treatment with silibinin (D/SB) and silibinin-loaded liposomes (D/LSB) in diabetic rats increased insulin levels by 1.7- and 1.6-fold, respectively, compared to the D group (Figure 1). While insulin levels in the C/L group remained at the control level, insulin levels in the C/SB and C/LSB groups were approximately 1.1-fold and 1.15-fold higher, respectively, compared to the C group (Figure 1).

### 3.3. Impact of Silibinin and Silibinin-Loaded Liposomes on Biochemical Parameters in Non-Diabetic and Diabetic Rats

**Figure 2:**
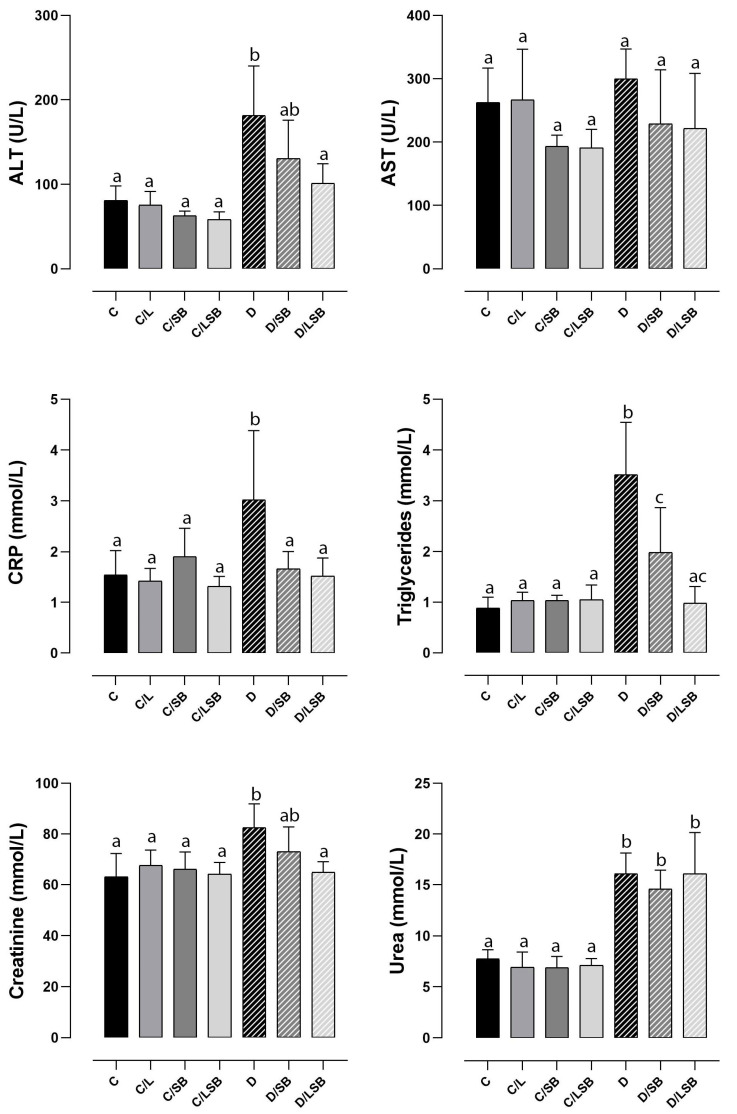
The impact of silibinin and silibinin-loaded liposomes on biochemical parameters in non-diabetic and diabetic rats. Serum concentrations of AST (aspartate aminotransferase), ALT (alanine aminotransferase), CRP (C-reactive protein), triglycerides, creatinine, and urea; C—non-diabetic group; C/L—non-diabetic group treated with liposomes; C/SB—non-diabetic group treated with silibinin; C/LSB—non-diabetic group treated with silibinin-loaded liposomes; D—diabetic group; D/SB—diabetic group treated with silibinin; D/LSB—diabetic group treated with silibinin-loaded liposomes. Results are expressed as means ± SD (N = 7 for non-diabetic groups; N = 8 for diabetic groups). Means followed by different letters (a, b, c) indicate significant differences at *p* < 0.05 (one-way ANOVA followed by Tukey’s multiple comparison test).

The serum levels of the markers for liver function, ALT, and AST are shown in Figure 2. The ALT level was significantly elevated (2.2-fold) in group D compared to the control (Figure 2). Treatment with silibinin in diabetic rats (D/SB) lowered ALT levels by 1.4-fold, while treatment with liposomal-encapsulated silibinin (D/LSB) reduced ALT levels by 1.8-fold compared to the D group (Figure 2). ALT levels in C/L, C/SB, and C/LSB showed no statistical difference compared to the control (Figure 2). AST levels showed a tendency to increase in diabetic rats and to decrease after administration of silibinin or silibinin-loaded liposomes, although no statistical significance was observed. In the C/L group, the AST level showed no difference compared to the C group, while in the control groups treated with silibinin (C/SB) and with silibinin-loaded liposomes (C/LSB), the AST level was slightly reduced but without statistical significance. The level of CRP was 1.8 times higher in the diabetic group (D) than in the C group (Figure 2). In both diabetic groups treated with silibinin (D/SB) and silibinin-loaded liposomes (D/LSB), CRP levels were comparable to the C group. In the control rats treated with plain liposomes (C/L), pure silibinin (C/SB), or silibinin-loaded liposomes (C/LSB), no statistically significant difference in CRP levels was observed compared to the control value (C) (Figure 2). The serum levels of triglycerides were increased fourfold in the D group compared to the control group (Figure 2). Treatment of diabetic rats with silibinin (D/SB) resulted in a significant decrease in triglycerides, which were 2.2-fold higher than in the control group, while administration of silibinin-loaded liposomes (D/LSB) reduced triglycerides to the level of the control group (C). The measurement of triglycerides in groups C/L, C/SB, and C/LSB showed no difference compared to the C group (Figure 2). Serum levels of the markers of kidney function, creatinine, and urea were significantly increased in the D group compared to the control group by 1.3- and 2-fold, respectively (Figure 2). Treatment of the diabetic rats with silibinin (D/SB) reduced creatinine levels by 1.2-fold, while administration of liposomes with silibinin (D/LSB) returned creatinine levels to the control level (C) (Figure 2). Treatment of the control rats with liposomes (C/L), pure silibinin (C/SB), or encapsulated silibinin (C/LSB) showed no difference in creatinine levels in comparison to the C group (Figure 2). Urea levels were reduced 1.1-fold in the diabetic group treated with silibinin (D/SB), while no difference was observed after treatment with silibinin-loaded liposomes (D/LSB) compared to the D group (Figure 2). In the control groups treated with liposomes (C/L), pure silibinin (C/SB), or silibinin-loaded liposomes (C/LSB), no statistical significance was found compared to the C group (Figure 2). According to the obtained results, silibinin-loaded liposomes decreased the levels of ALT, triglycerides, and creatinine in diabetic rats (D/LSB) more efficiently than pure silibinin (D/SB).

### 3.4. Histological Examination of the Pancreas following Treatment with Silibinin and Silibinin-Loaded Liposomes in Non-Diabetic and Diabetic Rats

**Figure 3:**
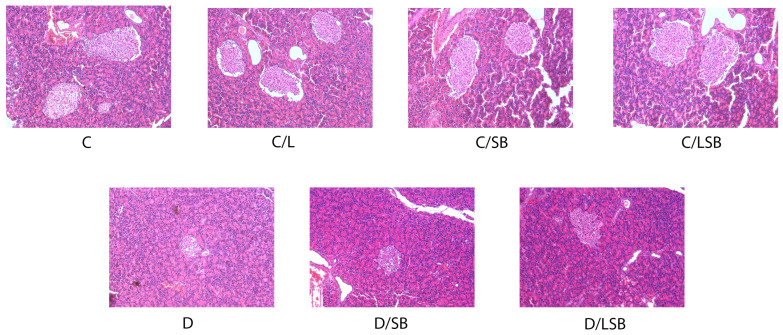
The effect of silibinin and silibinin-loaded liposomes on the histological changes in pancreatic tissue of non-diabetic and diabetic rats. Histological analysis of pancreatic islets assessed by hematoxylin and eosin staining of pancreas sections (magnification 20×); C—non-diabetic group; C/L—non-diabetic group treated with liposomes; C/SB—non-diabetic group treated with silibinin; C/LSB—non-diabetic group treated with silibinin-loaded liposomes; D—diabetic group; D/SB—diabetic group treated with silibinin; D/LSB—diabetic group treated with silibinin-loaded liposomes.

Hematoxylin and eosin-stained sections of the pancreas are shown in Figure 3. The findings reveal that the islets of Langerhans of the control rats consist of oval or elongated, compactly arranged cells in the form of dense strands (Figure 3, C). No discernible differences in the number, size, or structure of pancreatic islets were observed in control groups treated with pure liposomes, silibinin, or encapsulated silibinin (Figure 3, C/L, C/SB, and C/LSB, respectively). In contrast, the pancreas of diabetic rats exhibited a diminished number of islets, accompanied by condensed islet area and atrophy of the islets with increased vacuolization and cell loss (Figure 3, D). Treatment of diabetic rats with silibinin or silibinin-loaded liposomes resulted in a restoration of pancreatic architecture, assuming a near-control organization of islets (Figure 3, D/SB and D/LSB).

### 3.5. Effects of Silibinin and Silibinin-Loaded Liposomes on Insulin, Glucagon, and Glut2 in the Pancreatic Islets of Non-Diabetic and Diabetic Rats

**Figure 4:**
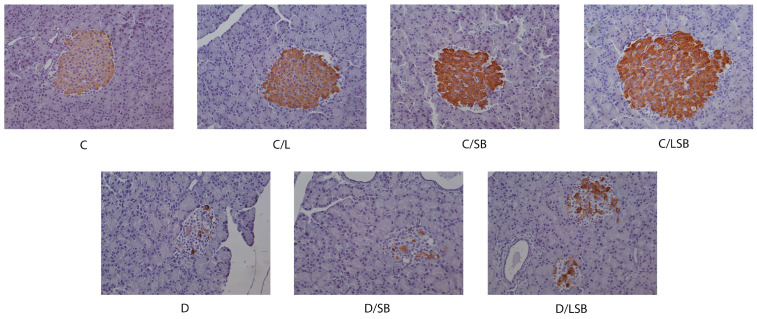
The impact of silibinin and silibinin-loaded liposomes on the presence and localization of insulin in the pancreatic islets of non-diabetic and diabetic rats. The immunohistochemical staining of pancreas sections with anti-insulin antibody showing brown insulin staining (DAB) of the beta cells in representative pancreatic islets (magnification 20×); C—non-diabetic group; C/L—non-diabetic group treated with liposomes; C/SB—non-diabetic group treated with silibinin; C/LSB—non-diabetic group treated with silibinin-loaded liposomes; D—diabetic group; D/SB—diabetic group treated with silibinin; D/LSB—diabetic group treated with silibinin-loaded liposomes.

**Figure 5:**
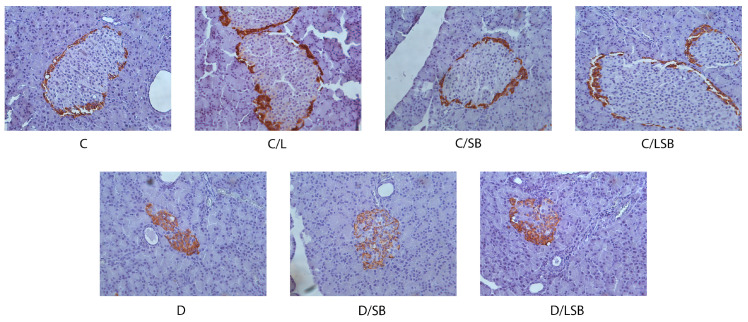
The presence and distribution of glucagon in the pancreatic islets of non-diabetic and diabetic rats under silibinin treatment. The immunohistochemical staining of pancreas sections with anti-glucagon antibody showing brown insulin staining (DAB) of the alpha cells in representative pancreatic islets (magnification 20×); C—non-diabetic group; C/L—non-diabetic group treated with liposomes; C/SB—non-diabetic group treated with silibinin; C/LSB—non-diabetic group treated with silibinin-loaded liposomes; D—diabetic group; D/SB—diabetic group treated with silibinin; D/LSB—diabetic group treated with silibinin-loaded liposomes.

The immunohistochemical evaluation of the presence of insulin and glucagon in the pancreatic islets is presented in Figure 4 and Figure 5, respectively. Immunostaining with anti-insulin antibody in the control group (C) and the control groups treated with liposomes (C/L), silibinin (C/SB), or silibinin-loaded liposomes (C/LSB) showed strong insulin staining of the beta-cells, which was evenly distributed over the islets (Figure 4). In contrast, the pancreatic islets of diabetic rats (D) showed a significantly reduced number of insulin-positive cells with disturbed composition (Figure 4). Treatment of diabetic rats with silibinin resulted in a slight increase in insulin-positive regions in the islets (D/SB), while administration of silibinin-loaded liposomes to diabetic rats considerably increased the zones of insulin-positive beta cells in the islets (D/LSB) (Figure 4).

**Figure 6:**
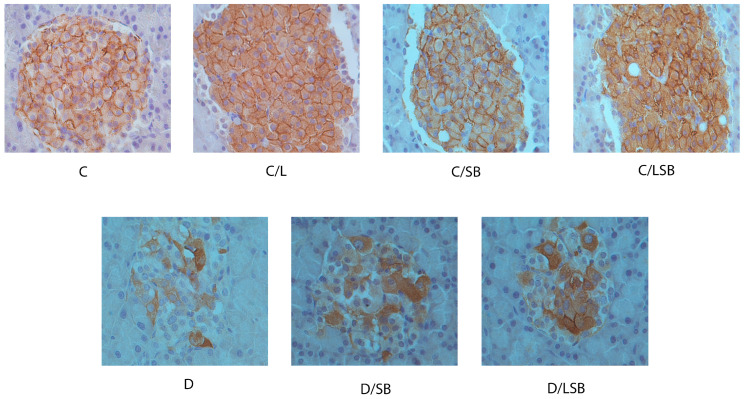
Silibinin and silibinin-loaded liposomes affect the presence and localization of GLUT2 in the pancreatic islets of diabetic rats. The immunohistochemical staining of pancreas sections with anti-GLUT2 antibody showing brown insulin staining (DAB) in representative pancreatic islets (magnification 40×); C—non-diabetic group; C/L—non-diabetic group treated with liposomes; C/SB—non-diabetic group treated with silibinin; C/LSB—non-diabetic group treated with silibinin-loaded liposomes; D—diabetic group; D/SB—diabetic group treated with silibinin; D/LSB—diabetic group treated with silibinin-loaded liposomes.

Immunostaining with an anti-glucagon antibody showed the peripheral distribution of alpha cells in the pancreatic islets of the control group (C) and the control groups treated with liposomes (C/L), silibinin (C/SB), or encapsulated silibinin (C/LSB) (Figure 5). In the diabetic group (D), the alpha cells stained with glucagon were centrally located in the islets. Administration of silibinin to diabetic rats (D/SB) (Figure 5) reduced the central distribution of glucagon-stained cells compared to group D, while treatment of diabetic rats with silibinin-loaded liposomes (D/LSB) significantly improved the peripheral localization of alpha cells compared to diabetic islets, but still with a significant deviation compared to control (C) (Figure 5). As shown in Figure 6, immunohistochemical staining with an anti-Glut2 antibody revealed the membrane localization of Glut2 in the cells of pancreatic islets of control rats (C) as well as in the control groups treated with liposomes (C/L), silibinin (C/SB), or liposome-encapsulated silibinin (C/LSB). Under diabetic conditions, a loss of membrane localization of Glut2 was observed, which was replaced by cytoplasmic localization of Glut2 (D). In the islets of diabetic rats treated with silibinin (D/SB), a partial recovery and an increase in membrane localization of Glut2 is observed, which was even more pronounced after treatment with silibinin-loaded liposomes (D/LSB).

### 3.6. Silibinin and Silibinin-Loaded Liposomes Reduce Collagen Deposition in the Pancreas of Diabetic Rats

**Figure 7:**
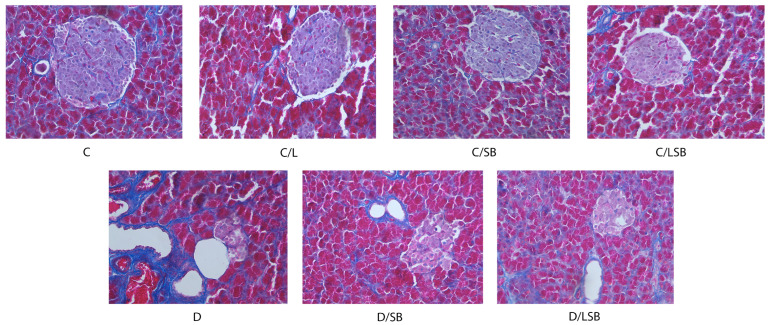
Silibinin and silibinin-loaded liposomes diminish collagen deposition in pancreatic tissue of diabetic rats. Masson trichrome staining showing collagen deposition in pancreas sections (blue: collagen, pink/violet: cytoplasm, and brown: nuclei) (magnification 20×); C—non-diabetic group; C/L—non-diabetic group treated with liposomes; C/SB—non-diabetic group treated with silibinin; C/LSB—non-diabetic group treated with silibinin-loaded liposomes; D—diabetic group; D/SB—diabetic group treated with silibinin; D/LSB—diabetic group treated with silibinin-loaded liposomes.

Masson’s trichrome staining of the pancreas for collagen fiber deposition is presented in Figure 7. As can be seen, collagen fibers were noted around the blood vessels, in the thin septa, and around the islets in the control group (C). Similar observations were made in the control groups treated with pure liposomes, pure silibinin, or silibinin-loaded liposomes (C/L, C/SB, and C/LSB, respectively). In the diabetic group (D), Masson staining showed an increase in collagen fiber deposition in the islets and around the pancreatic ducts and blood vessels. Administration of silibinin (D/SB) or encapsulated silibinin (D/LSB) to diabetic rats reduced collagen deposition in all pancreatic structures, whose morphology was more similar to that of the control group (Figure 7).

## 4. Discussion

Given the potential of silibinin as an antidiabetic agent, it is of crucial importance not only to evaluate its biological effects but also to optimize its bioavailability for diabetes treatment. To date, various delivery systems such as micronized form, phytosomes, or nanoparticles have been explored to improve the aqueous solubility and efficacy of orally administered silibinin or silymarin [19]. The micronized as well as phytosomal formulations of silymarin increased its water solubility and oral bioavailability compared to pure silymarin, along with its enhanced activity in stimulating lactation in healthy women [34] or improved hepatoprotective effects in a CCl4-induced hepatotoxicity rat model [35]. Engineered biopolymeric nanoparticles with a silibinin encapsulation efficiency of 92.11% significantly ameliorated hyperglycemia, as well as oxidative stress parameters in the serum and liver of STZ-diabetic rats due to improved silibinin dissolution and passive transport [36]. These results prompted us to investigate how to improve the antidiabetic efficacy of silibinin by utilizing the existing knowledge on liposomal vesicles [25], which was tested in STZ-diabetic rats. Liposomal vesicles are often used for the controlled delivery of drugs, proteins, polyphenols, vitamins, flavors, and antioxidants [21,22,25,27]. Among other carriers, liposomal particles have better fluidity and mobility through the native plasma membrane due to their three-dimensional and spherical structure and are biocompatible due to their bilayer, which corresponds to a biological membrane [23,37]. Characterization of the prepared silibinin-loaded liposomes revealed a high encapsulation efficiency and stability of the system, which may greatly contribute to prolonged in vivo retention of the encapsulated substance and its bioavailability. The high encapsulation efficiency of silibinin in phospholipid liposomes (>96%) in our study is consistent with literature data, according to which the encapsulation efficiency of resveratrol (a phenolic compound from the plants as well) in liposomes was ~97% [27]. The achieved encapsulation efficiency can be explained by the lipid composition of the prepared liposomes loaded with silibinin. In the present study, the liposomal vesicles contain only phospholipids (without the addition of sterols), which makes the liposomal bilayer stiffer and, at the same time, prevents the leakage of silibinin. Additionally, the particle size of liposomes is an essential and relevant parameter for the stability, biodistribution, and release of bioactive substances [25]. The significantly larger vesicle size of the encapsulated silibinin compared to the literature data (e.g., the size of liposomes with resveratrol prepared by the proliposome method was ~500 nm [27]) can be explained by the location where the silibinin was entrapped in the liposomes. Namely, as a hydrophobic compound, silibinin can only be entrapped within the liposomal bilayer (between the phospholipid tails), which leads to the formation of a wider inter-lipid space, expansion of the membrane, and increased size of liposomes. Moreover, the measured PDI value of silibinin-loaded liposomes indicates a moderately dispersed distribution [38]. The obtained results of zeta potential are in agreement with the literature data, according to which the liposomes with resveratrol prepared by the proliposome technique showed a zeta potential of ~−25 mV [27]. The negative and high value of the zeta potential, which was also measured in the system of liposomes with silibinin, provides good electrostatic stabilization, which reduces the possibility of occurrence of vesicle aggregation and fusion [27]. Surface charge is also responsible for the behavior of vesicles and encapsulated content and its elimination in vivo. Therefore, anionic vesicles (as in the case of the obtained silibinin-loaded liposomes) strongly interact with reticuloendothelial cells by intercepting endothelial cells and blood resident macrophages, thus preventing/decreasing the elimination of liposomes. On the other hand, cationic particles can be rapidly eliminated via non-specific interactions, i.e., adsorption to the anionic surface of the blood vessel walls, and degraded by specialized cells of the reticuloendothelial system [39]. It is expected that the achieved high encapsulation efficiency and stability of the silibinin-loaded liposomes could contribute to a controlled release of silibinin and, thus, to its better bioavailability. The mobility of liposomal vesicles is a key parameter for the delivery of bioactive components and is correlated to the interactions between liposomes and entrapped substances and the microstructure of the system [40]. In addition, changes in liposomal mobility were attributed to the mechanical rigidity or the ability of the vesicles to deform. Hence, softer and more fluid liposomal particles, which, apart from the phospholipids, contain sterols, such as cholesterol, exhibit higher mobility. Nevertheless, due to the negative effects of cholesterol on already existing disorders related to diabetes, the prepared silibinin-loaded liposomes contained only phospholipids and can be rigid, explaining their lower mobility [40].

Our study revealed that silibinin, both pure and encapsulated in liposomal particles, improves levels of glucose, insulin, and GlyHb in the circulation of diabetic rats. Accumulating experimental and clinical data also suggest that silymarin and its constituents have hypoglycemic effects by lowering blood glucose levels and increasing insulin secretion [41]. Oral administration of silibinin (40 or 80 mg/kg/daily for 4 weeks) restored hyperglycemia and hyperlipidemia in STZ-induced diabetic rats [17]. Accordingly, oral gavage of 200 mg/kg silibinin for 10 weeks to high-fat diet (HFD) C57BL/6J mice improved insulin secretion and the control of blood glucose [42]. Silymarin (80 mg/kg, orally for 21 days) was also shown to reduce fasting blood sugar levels in STZ-induced diabetic rats [43]. A meta-analysis of data encompassing five randomized controlled trials conducted on 270 patients with T2D revealed that the administration of silymarin might improve glycemic control by reducing fasting blood glucose and GlyHb levels [18]. Silibinin has been reported to improve glycemic control by reducing gluconeogenesis and glucose-6-phosphatase activity in the liver [44] by triggering the gut–brain–liver axis [16]. Another study showed that silibinin may reduce both basal and insulin-stimulated glucose uptake in 3T3-L1 cells by directly reducing glucose binding to GLUT4 due to the same binding sites on GLUT4 shared by silibinin and glucose [45]. Considering that glucose homeostasis depends on the insulin-controlled balance between hepatic glucose production and glucose utilization in peripheral tissues, the above findings strongly suggest multi-targeted effects of silibinin on glucose homeostasis mechanisms [15]. Our results further suggest that the beneficial effects of silibinin on glycemic control could be more pronounced by its encapsulation in liposomes. While the encapsulation of silibinin demonstrated similar effects in reducing GlyHb levels and increasing insulin availability compared to pure silibinin, a notably superior impact on glucose levels was observed in diabetic animals treated with silibinin-loaded liposomes.

The documented control of blood glucose levels mediated by silibinin may be partly due to the protection of pancreatic islet cells from death and dysfunction. According to previous data, diabetic rats treated with 80 mg/kg of silibinin for 28 days showed improved histological structure of the pancreas [17]. Administration of silibinin (50 mg/kg/day/intramuscular) for 8 weeks reversed STZ-induced apoptosis of beta cells, likely through upregulation of Silent information regulator 1 (Sirt1), which is thought to promote glucose-stimulated insulin secretion [46]. In pancreatectomized rats, silymarin (200 mg/kg/orally) stimulated gene expression of pancreatic and duodenal homeobox 1 (Pdx1) factor involved in pancreatic growth and insulin gene expression and NK6 homeobox 1 (NKx6.1) protein responsible for neogenesis, differentiation, and maintenance of beta cells, which was accompanied by enhanced insulin gene expression along with improved beta-cell proliferation [47,48]. Our study further confirms the important role of silibinin in protecting islet cells from STZ-induced damage. Histological examination of the pancreas after treatment with silibinin and silibinin-loaded liposomes showed a silibinin-mediated reduction of STZ-induced damage in diabetic rats. While histological assessment revealed no discernible beneficial effect of silibinin encapsulation, immunohistochemical analysis of pancreatic islets clearly showed that silibinin-loaded liposomes were superior in protecting islet architecture than pure silibinin. In particular, an increase in the number of insulin-positive cells, an improved distribution of alpha cells, as well as a partial recovery, and an increase in membrane localization of Glut2 were observed after treatment with silibinin-loaded liposomes compared to pure silibinin. The explanation can lay in the fact that the liposome phospholipid bilayer, which is similar to the cell membrane, provides an enhanced transfer into cells of the islets of Langerhans and, consequently, a higher concentration of the bioactive compound, i.e., silibinin in the cells, resulting in a more intense effect on insulin availability [49]. These findings suggest that the liposomal formulation can ensure an effective concentration of silibinin inside the cells and enhance the protective effect of silibinin on the preservation of the structure and function of the pancreatic islets, which in turn provides better control of glycemia and lipid parameters.

Glucose and lipid metabolism are linked in numerous ways, and more recent data indicate that hypertriglyceridemia and low HDL may not only be the consequence but also the cause of impaired glucose metabolism [50]. Previous studies revealed that the use of silibinin can contribute to a better control of lipid metabolism. In experiments with zebrafish, the administration of 5 µM silibinin led to an inhibition of lipid accumulation, accompanied by a reduction in the expression of adipogenic factors such as fatty acid-binding protein 4 (FABP4), peroxisome proliferator-activated receptor gamma (PPARγ), and CCAAT-binding protein alpha (C/EBPα), as well as a reduction in triglyceride levels [51]. Treatment of diabetic rats with silibinin (80 mg/kg for 28 days) decreased total cholesterol, triglyceride, and LDL and increased HDL levels in serum as compared to non-treated diabetic rats [17]. The present study demonstrated the advantage of encapsulating silibinin in liposomes concerning its impact on triglyceride levels in diabetic conditions. While the administration of silibinin to diabetic rats led to a reduction in triglyceride levels, treatment with silibinin-loaded liposomes significantly enhanced this effect, as triglyceride levels were maintained at the control level. The liposomal bilayer is flexible so that liposomal particles can penetrate the capillary endothelium of the liver and be taken up by hepatocytes [52]. The aforementioned penetration and reaching the hepatocytes, therefore, enable silibinin to have a better influence on the metabolism of lipids, especially triglycerides. This finding is of particular importance when one considers that a disturbed glucose and lipid metabolism ultimately leads to diabetic complications.

Various liver abnormalities, including non-alcoholic fatty liver disease (NAFLD) characterized by hepatic deposition of triglycerides, are associated with diabetes [53]. NAFLD includes increased lipogenesis, increased triglyceride vacuoles, ballooning, and necrosis of hepatocytes, together with inflammatory cell infiltration and fibrosis [54]. Silibinin is frequently used in the clinic due to its liver-protective effect through the suppression of inflammation, apoptosis, oxidative damage, and fibrosis [15]. The administration of silibinin to diabetic rats in our study significantly lowered ALT levels, while treatment with encapsulated silibinin in liposomes further enhanced this effect, reducing ALT levels by almost twofold compared to diabetic rats. One study reported that the silymarin-loaded liposomes showed a stronger hepatoprotective activity and significantly decreased ALT concentration in the serum in comparison to orally administered non-encapsulated silymarin at the same doses due to increased intestinal absorption and penetration within hepatocytes [55]. Moreover, silymarin has been demonstrated to reduce serum creatinine levels in rats and to ameliorate diabetes-induced nephropathy through antioxidant effects [56,57]. Administration of silymarin (140 mg/daily for 3 months) to T2D patients with overt nephropathy (randomized, double-blind, placebo-controlled trial) reduced urinary excretion of albumin, TNF-α, and malondialdehyde (MDA; an oxidative stress marker) suggesting its renoprotective effect [58]. Administration of silymarin (50 and 100 mg/kg/day for 30 days) to alloxan-induced diabetic rats reduced creatinine levels and lowered protein damage in the liver and pancreas [59]. Our findings showed that silibinin reduced creatinine levels by 1.2-fold, while the administration of encapsulated silibinin returned creatinine levels to the control level, strongly suggesting that the liposomal formulation may enhance the renoprotective effect of silibinin in diabetic conditions. Significant differences between the impact of non-encapsulated herbal compounds and their liposomal encapsulated parallels on renal function parameters, such as creatinine levels and renal cytoarchitecture, are shown in several research studies, supporting the conclusion of higher bioavailability and the impact of liposomal encapsulated compounds on the maintenance of renal function [60].

Dysregulation of signaling and metabolism in the liver predisposes individuals to T2D and/or NAFLD so that certain liver-derived biomarkers such as CRP, alpha-hydroxybutyrate, or fetuin-A can be used for the diagnosis and prognosis of diabetes and its complications [61]. CRP is secreted by the liver in response to a variety of inflammatory cytokines, rises quickly in response to infection, trauma, or inflammation, and falls just as quickly when the condition subsides [62]. Therefore, CRP is normally used to monitor various inflammatory conditions. In the present study, CRP levels in diabetic rats were almost doubled compared to the control group, while the treatment with silibinin or silibinin-loaded liposomes reduced CRP to control levels. Our findings are in correlation with the anti-inflammatory effects of silymarin observed in the diabetic liver and experimental inflammatory bowel disease [18]. Preclinical and clinical studies have shown the suppressive effect of silymarin on the release of pro-inflammatory cytokines such as TNF-α, IL-1β, IL-6, and IL-12 [63]. As they play a crucial role in the control of glucose homeostasis, any irregular change in pro-inflammatory cytokines could reduce insulin sensitivity and contribute to IR. In addition, infiltration of cells producing pro-inflammatory cytokines leads to beta-cell failure [59]. The attenuation of the inflammatory response caused by silymarin through inhibition of NF-kB target genes could be the basis for its positive effect against IR and liver damage [63,64]. Thus, silymarin suppresses TNF-α- and IL-1β-induced formation of nitric oxide and expression of inducible nitric oxide synthase (iNOS) in beta cells by modulating NF-κB activity and signaling of extracellular signal-regulated kinase1/2 (ERK1/2), thereby preventing beta-cell destruction [63].

In contrast to acute inflammatory reactions, which are characterized by rapidly subsiding vascular changes, neutrophilic inflammation, and edema, chronic inflammation leads to fibrosis, which is characterized by overgrowth, hardening, or scarring of various tissues due to excessive deposition of extracellular matrix components, including collagen [65]. Among other factors, acute-phase proteins and cytokines are considered to be important regulators of fibrosis. Pancreatic fibrosis results from the excessive deposition of collagen fibers during multiple necrosis to repair damaged pancreatic tissue [66]. In our study, the increase in collagen fiber deposition in pancreatic islets of diabetic rats was reduced by the administration of silibinin or silibinin-loaded liposomes so that the morphology of the pancreas became more similar to that of the control group. This is consistent with results suggesting anti-inflammatory and anti-fibrotic properties of silymarin [10,18]. Silymarin was found to suppress the expression of pro-fibrogenic procollagen alpha 1 (I) and tissue inhibitor of metalloproteinase-1 (TIMP-1), most likely by decreasing the expression of transforming growth factor-beta 1 (TGF-β1) in rats with biliary fibrosis [67]. In another study, silymarin (50 mg/weight, one month) inhibited the progression of fibrosis in the early stages of liver injury in the CCl4-induced liver fibrosis model in rats [68]. Furthermore, treatment with silibinin (105 mg/kg/day for 8 weeks) significantly alleviated liver inflammation, fibrosis, and steatosis in C57BL/6 mice with non-alcoholic steatohepatitis [69]. Our results show for the first time that silibinin protects pancreatic islet cells from damage by reducing the deposition of collagen fibers, which strongly supports the anti-inflammatory effects of silibinin. On the other hand, the size of the liposome particles may explain the lack of a statistically significant difference between the effect of pure silibinin and its encapsulated counterpart on collagen fiber deposition in this study. According to the literature data, the accumulation of nanoparticles with a size of ~100 nm is expected in the pancreas as their diameter is small enough to extravasate from the blood, increase permeability, and improve the retention effect [70]. However, silibinin-loaded liposomes had a 20-fold larger size (~2000 nm, Table S1), which could be significantly reduced by additional procedures such as extrusion or sonication. With regard to the smaller size, small unilamellar particles have a significantly higher specific surface area, which additionally contributes to the increased absorption and release of the encapsulated components. However, the reduction in vesicle size can lead to an increase in PDI and a reduction in zeta potential, as well as destabilization of the liposomal system and agglomeration and fusion of the particles under storage conditions [27]. Since the animals received silibinin-loaded liposomes for 4 weeks, liposome stability was of great importance. Therefore, the stability of the non-sonicated liposomes (multilamellar vesicles used in the present study) and sonicated liposomes (small unilamellar vesicles) was monitored for 60 days [71]. The data confirmed a significantly lower stability in the case of small silibinin-loaded liposomes (increased values of size and PDI and decreased values of zeta potential and mobility). Thus, more stable multilamellar liposomes with silibinin were selected for further experiments on animal models. Additionally, during the sonication of liposomal particles by employing the ultrasound probe (because ultrasound waves in the ultrasound bath could not provide a significant size reduction in the case of liposomes with silibinin), the probe debris remains in the liposomal suspension, which can be visible after the centrifugation.

## 5. Conclusions

The antidiabetic effects of silibinin encapsulated in liposomes proved to be better than those of pure silibinin. The high encapsulation efficiency of silibinin in phospholipid liposomes and its sustained stability (due to a higher absolute value of zeta potential) in our study suggest a promising approach to improve the utility of silibinin in the treatment of diabetes. The present study showed that silibinin lowered glucose and GlyHb levels in diabetic rats and improved insulin levels, as well as liver and kidney function parameters. In addition, silibinin restored islet cell architecture, which was disrupted under diabetic conditions, and showed an anti-inflammatory effect, as evidenced by a decrease in blood CRP levels and reduced deposition of collagen fibers in the islets of diabetic rats. Moreover, silibinin encapsulated in liposomes showed a significantly better effect than pure silibinin on glucose, ALT, triglyceride, and creatinine levels, on beta-cell mass, and the distribution of alpha cells and Glut2 receptors in the islets of diabetic rats. Silibinin-loaded liposomes are, therefore, a promising way to increase the efficacy of silibinin in combating diabetes and its complications and to serve as adjunctive therapeutics in the control of diabetes.

## Data Availability

The original contributions presented in the study are included in the article/Supplementary Material, further inquiries can be directed to the corresponding author.

## Supplementary Materials

The following supporting information can be downloaded at: https://www.mdpi.com/article/10.3390/pharmaceutics16060801/s1, Table S1. Encapsulation efficiency (EE), vesicle size, polydispersity index (PDI), zeta potential, and mobility of silibinin-loaded liposomes; Figure S1. Graphical presentation of silibinin-loaded liposome (A) size distribution by volume, (B) size distribution by intensity, (C) zeta potential distribution, and (D) mobility distribution measured using photon correlation spectroscopy (dynamic light scattering).

## Abbreviations

Type 1 diabetes (T1D); type 2 diabetes (T2D); insulin resistance (IR); interleukin-1 beta (IL-1β); interleukin 6 and 12 (IL-6 and IL-12); tumor necrosis factor alpha (TNFα); interferon gamma (IFNγ); C-reactive protein (CRP); polydispersity index (PDI); carboxymethyl-celulose (CMC); nicotinamide (NA), streptozotocin (STZ), 3,3′-diaminobenzidine (DAB); silibinin (SB); hemoglobin (Hb); glycated hemoglobin (GlyHb); blood urea nitrogen (BUN); glucose receptor 2 and 4 (Glut2 and GLUT4); tissue inhibitor of metalloproteinase-1 (TIMP-1); transforming growth factor-beta 1 (TGF-1); carbon tetrachloride (CCl4); high-fat diet (HFD); silent information regulator 1 (Sirt1); duodenal homeobox 1 (Pdx1); NK6 homeobox 1 (NKx6.1); fatty acid-binding protein 4 (FABP4); peroxisome proliferator-activated receptor gamma (PPARγ); CCAAT-binding protein alpha (C/EBPα); low-density lipoprotein (LDL); high-density lipoprotein (HDL); non-alcoholic fatty liver disease (NAFLD); inducible nitric oxide synthase (iNOS); extracellular signal-regulated kinase1/2 (ERK1/2); nuclear factor kappa B (NF-κB); alanine aminotransferase (ALT); glutamic pyruvic transaminase (GPT); aspartate aminotransferase (AST); glutamic oxalacetic transaminase (GOT); international federation of clinical chemistry and Laboratory Medicine (IFCC).
