# Supplementary material for: Liposome Encapsulation Enhances the Antidiabetic Efficacy of Silibinin
Source: Pharmaceutics. 2024 Jun 13;16(6):801. doi: 10.3390/pharmaceutics16060801 (PMC11207473; doi:10.3390/pharmaceutics16060801)
Supplement: Supplementary file 1 [file pharmaceutics-16-00801-s001.zip › pharmaceutics-3036366-supplementary.pdf]

**Table S1.** Encapsulation efficiency (EE), vesicle size, polydispersity index (PDI), zeta potential, and mobility of silibinin-loaded liposomes measured immediately after the preparation.

| Parameter | Value |
|---|---|
| EE [%] | >96 |
| vesicle size [nm] | 2024.7±22.1 |
| PDI | 0.323±0.025 |
| zeta potential [mV] | -26.2±0.6 |
| mobility [μmcm/Vs] | -2.06±0.05 |

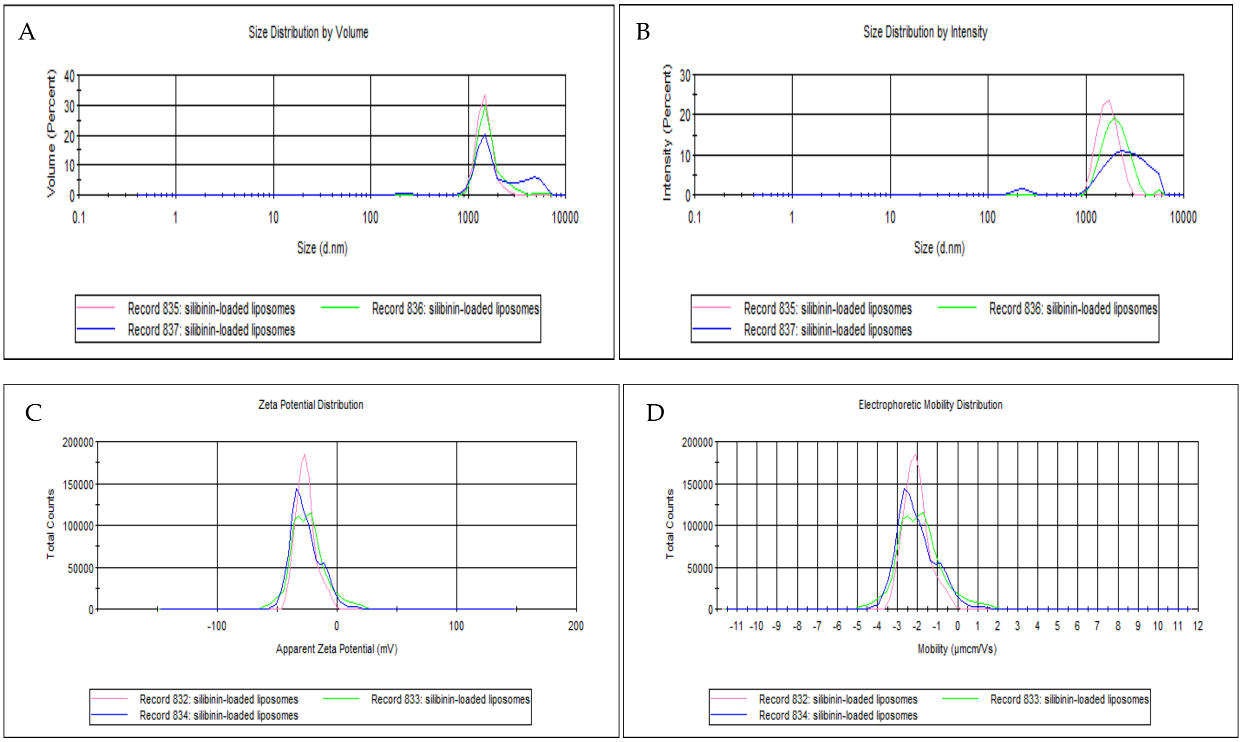


**Figure S1.** Graphical presentation of silibinin-loaded liposome (A) size distribution by volume, (B) size distribution by intensity, (C) zeta potential distribution, and (D) mobility distribution measured using photon correlation spectroscopy (dynamic light scattering)
